# The role of air breathing in exhaustive exercise recovery in Atlantic tarpon (*Megalops atlanticus)*

**DOI:** 10.1093/conphys/coaf089

**Published:** 2025-12-24

**Authors:** Leighann Martin, Angelina M Dichiera, Andrew J Esbaugh

**Affiliations:** Marine Science Institute, University of Texas at Austin, Port Aransas, TX 78373, USA; College William & Mary, Virginia Institute of Marine Science, Gloucester Point, VA 23062, USA; Marine Science Institute, University of Texas at Austin, Port Aransas, TX 78373, USA

**Keywords:** Anaerobic metabolism, catch and release angling, haemoglobin, recreational fishing, Root effect

## Abstract

Atlantic tarpon (*Megalops atlanticus*) are prized sportfish found through the Gulf of Mexico/America. Atlantic tarpon populations are also considered vulnerable to extinction, and thus many of the recreational fisheries targeting Atlantic tarpon in North America are limited to catch-and-release (CAR). While CAR procedures are common and effective means of protecting recreational sportfish species, it is important to recognize that species-specific traits can impact their efficacy. Here, we sought to explore the importance of Atlantic tarpon air-breathing behaviour in the context of recovery from exercise, which may impact their vulnerability to CAR angling events. A first series of experiments demonstrated that Atlantic tarpon increased air breathing rate following exposure to hypoxia—reinforcing their status as a facultative air-breather—but not following exhaustive exercise. A second series of experiments assessed whether the recovery of biochemical indicators of exhaustive exercise stress in the white muscle and plasma would be impacted by restricted air access during recovery. For fish with access to air, normal patterns of exhaustive exercise were noted in the plasma and white muscle with the exception that haematological parameters were unaffected by exercise. Access to air resulted in no significant differences in recovery profiles at the 1-h time point. Interestingly, exercise resulted in a significant and sustained reduction in red blood cell pH, which coincided with a significant impairment in oxygen binding affinity at higher oxygen partial pressures, possibly explaining why air-breathing behaviour is not beneficial during exercise recovery. Overall, these data suggest that Atlantic tarpon conform to typical patterns of exercise recovery in fishes and that no special consideration are required with respect to CAR angling.

## Introduction

Recreational fisheries in the Gulf of Mexico/America (GoMA) have been estimated to provide in excess of $7 billion annually to the United States, and sportfish species like Atlantic tarpon (*Megalops atlanticus*) are thought to be major contributors (National Marine Fisheries Service, 2018). These “Silver Kings” are highly sought after by anglers; tarpon’s massive size, acrobatics and prolonged fight times have made them a prized saltwater catch. In the 1800s, tarpon were abundant throughout the Gulf of Mexico, especially around the Texas coastal bend where many flocked for tarpon fishing. However, in the 1950s anglers claimed tarpon populations abruptly declined, but the reasons were unknown (Ault and Luo, 2013; Adams *et al.*, 2014). Currently, Atlantic tarpon are designated as a vulnerable species by the IUCN Red List (IUCN, 2025). In response to declining populations, decision-makers began to prioritize catch-and-release (CAR) practices in Texas—and many other regions of the GoMA—in an effort to protect and rebuild their populations (Adams *et al.*, 2014).

CAR angling is widely considered a useful conservation tool for fisheries managers to ensure that fish are returned to the wild and can repopulate the habitat. Nonetheless, evidence has consistently shown that CAR angling is an intense stressor for fish, which leads to both physiological exhaustion and a cascade of cellular stress responses with varying organismal outcomes (Cooke *et al.*, 2013). Compounding factors such as the type of hook, fishing line, fight times, handling and air exposure all impact the stress experienced by a fish (Cooke *et al.*, 2013). Exhaustive exercise primarily relies on anaerobic metabolism, which results in fish utilizing energy stores like ATP and phosphocreatine while raising the concentration of lactate in glycolytic skeletal (i.e. white) muscle (Kieffer, 2000). When released back into the environment after bouts of prolonged intense exercise, fish can be more susceptible to predation presumably due to their state of exhaustion (Danylchuk *et al.*, 2007). Observations of increased plasma and muscle lactate, reduced nucleoside triphosphate (NTP) concentrations as well as elevated haematocrit have been observed following angling events in Indo-Pacific tarpon (*M. cyprinoides*) (Wells *et al.*, 2003); however, little information is available for Atlantic tarpon.

While decades of research has helped refine the best practice protocols for CAR recreational angling, species-specific considerations remain integral to ensure the best chance for individual survival (Cooke and Suski, 2005). For example, several shark species are particularly susceptible to CAR practices owing to their reliance on ram ventilation, such as the hammerhead shark (Shiffman and Hammerschlag, 2016). These animals are at a unique risk of suffocation following exhaustive exercise because they must maintain forward momentum to obtain oxygen and clear lactate. Similar concerns may also apply to air-breathing fishes, such as Atlantic tarpon, if they are heavily reliant on aerial respiration. In fact, observations from Indo-Pacific tarpon suggest that this species may utilize air breathing during exercise recovery, as evidenced by the fact that plasma lactate recovered more quickly from an angling event when the animals were given air access (Wells *et al.*, 2003). The aerial dependence of air-breathing fish can vary widely, but species are broadly classified as either facultative or obligate (Graham, 1997). Facultative air-breathers utilize air breathing to augment branchial respiration during periods of need, but do not require it for normal survival. Conversely, obligate air-breathers require access to the air for baseline survival as their gills are not effective gas transport organs. One of the most well-known obligate air-breathers is the *Arapima gigas*, which has been shown to drown in less than 10 min without access to air (Val and Almeida-Val, 1995).

Tarpon (*Megalops* spp.) are the only known genus of air-breathing marine fishes, and the full extent of their aerial dependence remains an open question. This makes it difficult to properly account for air-breathing dependence in tarpon CAR protocols ostensibly designed for species conservation. The available data for the Indo-Pacific tarpon (*M. cyprinoides*) suggest this species is a facultative air-breather (Seymour *et al.*, 2004). Yet, forced submersion experiments on Atlantic tarpon (*M. atlanticus*) suggested that air breathing in this species was obligate (Breder and Shlaifer, 1940). More recently, Geiger *et al.* (2000) used an oxygen draw-down method to demonstrate higher air-breathing frequency at lower PO_2_, which is indicative of a facultative response. But this trend was only observed at low test temperatures (≤22°C), which makes the findings somewhat ambiguous. On this background, the purpose of this study was to explore the importance of air breathing to recovery from exhaustive exercise in the Atlantic tarpon with the intent to inform on species-specific CAR protocol design. More specifically, this study had three goals: (i) to use hypoxia exposure to validate that Atlantic tarpon display facultative air breathing behaviour, (ii) to determine if exhaustive exercise stimulates air breathing during recovery, and (iii) to assess the importance of air breathing for lactate clearance and the recovery of muscle and plasma metabolites following exhaustive exercise.

## Materials and Methods

### Field sites and fish collection

All experiments were approved by the University of Texas at Austin’s Institutional Animal Care and Use Committee (IACUC). Small juvenile Atlantic tarpon (<15 cm; *N* = 6) were caught using a 4′ cast net in freshwater creeks in Port Aransas, Texas (27.819030 N, 97.073100 W) from October 2017 to January 2018 over three trips. Once caught, fish were transported in aerated freshwater to the Fisheries and Mariculture Lab (FAML) at the University of Texas Marine Science Institute (Port Aransas, TX). Larger juvenile tarpon (>30 cm; mass ± S.E.M = 348.0 ± 85.7 g; *N* = 22) were caught using a seine net at a brackish water channel in Aransas Pass, TX (27.891130 N, 97.151890 W) from February 2018 to August 2019 over eight collection trips. Fish were placed in a 500-L transportation tank filled with freshwater, transported to FAML, and placed in 1000-L husbandry tanks for at least 48 h prior to experimentation. Note that spring 2020 collections were disrupted by covid-19, which resulted in lower sample sizes for one experimental time point.

### Husbandry

Small juveniles were housed in two 40-L aquaria (3 per tank) with recirculating and aerated freshwater (dechlorinated Port Aransas tap water) maintained at 25°C. Ammonia was controlled using biofiltration, and a 50% water change was performed once a week. Small juveniles were allowed to acclimate to the holding facilities for approximately 1-month prior to the onset of behavioural experimentation. Fish were initially fed to satiation with freshly cut and peeled shrimp, and subsequently transferred to dry food pellets (Aquamax, Purina). Large juveniles were housed in 1000 L tanks containing aerated freshwater and filtration and maintained at 26–27°C. Larger fish were kept at low density (1–3 per tank) and fed fresh shrimp until satiation every other day. For two fish, temperature acclimation was needed as the environmental temperature was approximately 21–22°C. In these cases, the temperature was changed at 1°C per day until 26°C after which the fish were held for at least 48 h prior to experimentation. Food was withheld from all fish for 24 h prior to experimentation.

### Air-breathing experiments

Small juvenile tarpon were used to assess whether air breathing was obligate or facultative in two experimental series. Series 1 measured the frequency of air breathing over 15-min intervals of declining oxygen saturation (% O_2_ saturation: 100, 80, 60, 40, 20 and 10). A recirculating system with a central header tank supplied water to two 5-L observation chambers. An automated oxygen control system consisting of an electrode and gas solenoid (Loligo Systems) bubbled nitrogen gas into the header tank to displace oxygen as needed. Each observation chamber held a single juvenile tarpon, and fish were acclimated to the chamber for 12 h at normoxia prior to experimentation. A GoPro Hero 4 recorded video of each 15-min interval. Videos were manually analyzed for air-breathing frequency, which was defined by surfacing, gulping air, and a subsequent bubble release from the operculum.

Series 2 used the same juvenile fish as in Series 1 (*N* = 6), but investigated a longer exposure interval. This experiment again assessed the effects of normoxia and hypoxia on air-breathing behaviour, but also introduced exhaustive exercise as an additional driver of air breathing under both oxygen availability scenarios. Tarpon were first filmed for 1 h under normoxia or hypoxia (20% air saturation) to achieve a pre-exhaustive exercise control. Fish were then removed from the observation tank and chased to exhaustion in normoxia using a standard chase protocol (Turner *et al.*, 1983; Wood, 1991; Roche *et al.*, 2013; Ackerly and Esbaugh, 2020), which used a pinch tail method to chase fish for 3 min or until exhausted. Exhaustion was defined as when the fish lost equilibrium for at least 3 s. Fish were then returned to the observation chamber and filmed for 1 h in either normoxia or hypoxia. Note that air exposure was not included as part of the chase protocol. Also, note that the same six fish were used for both oxygen saturations, which was necessitated by the limited supply of animals. All fish were provided at least 7 days recovery between experimentation, to allow full recovery from exercise stress (Milligan, 1996). The fish also successfully fed between experiments, further suggesting they had recovered from the experimental stress.

### Exhaustive exercise and recovery experiments

Large juvenile tarpon (mass = 348.0 ± 85.7 g; *N* = 22) were utilized to explore the importance of air breathing on recovery profiles of exercise metabolites following exhaustive exercise. Fish were chased using a scaled-up version of the protocol described above. In this case, a single fish was chased by hand or net for 5 min, or until exhaustion. As above, air exposure was not included in the chase protocol. Tarpon were then transferred to a 40-L recovery chamber and allowed to recover for 1 and 4 h with or without access to air. A no-recovery treatment was sampled immediately post-exercise, and a no-exercise treatment was performed by placing fish in recovery chambers with air access for 36 h. The sample sizes were as follows: no exercise = 5; no recovery = 5; 1 h with air = 5; 1 h no air = 5 and 4 h with air = 2. Note that the small sample size in 4-h recovery treatments was due to covid-19-related cancellation of our final field season. After each treatment, tarpon were anaesthetized by adding buffered MS-222 (250 mg/L MS-222; 500 mg/L NaHCO_3_) to the recovery chamber through a hole in the lid, euthanized by spinal transection, and sampled for blood and white muscle. Note that this procedure was specifically designed in accordance with prior work demonstrating the efficacy of caudal puncture for blood pH determinations (Montgomery *et al.*, 2019; Negrete Jr *et al.*, 2022; Martin *et al.*, 2023), as well as muscle metabolites (Martin *et al.*, 2023; Negrete Jr *et al.*, 2024). Blood was collected using a 22-gauge heparinized needle by caudal puncture, and white muscle was dissected by scalpel from the left side of the body anterior to the dorsal fin. Blood samples were kept on ice until analyses (minutes), and white muscle was flash frozen in liquid N_2_.

### Oxygen equilibrium curves

Frozen red blood cell samples from no exercise and exercised no recovery fish (*n* = 4 per treatment) were used to develop hemolysates for determination of oxygen equilibrium curves (OEC), using previously described procedures (Negrete Jr *et al.*, 2022). Briefly, 12 μl of thawed and lysed red blood cells were diluted to a final volume of 40 μl in pH specific buffer (final concentrations: 10 mM KCl, 100 mM HEPES, 10 mM ascorbic acid; buffer pH 6.95, 7.3 and 8.0 with HCl/NaOH). Note that hemolysates were not stripped as we wanted to assess the simultaneous effects of pH and endogenous nucleoside triphosphate (NTP) level. Each sample had two lysates, a pH 8 (7.91 ± 0.01) and a treatment pH lysate (no exercise = 7.27 ± 0.01; no recovery = 6.93 ± 0.02). The pH of each lysate was verified using a micro-sample combination electrode (Accumet). The pH 8 lysate was used to develop the maximum saturation range for oxygen at alkaline pH, which was required to account for the Root effect of teleost haemoglobins. For measurement, 1 μl of each lysate was loaded into the measurement cell of a Loligo Systems Blood Oxygen Binding System (BOBS) set to 26°C. The sample was then exposed to 21% oxygen balanced with nitrogen for 10 min to allow sample to equilibrate and stabilize after which the sample was exposed to 0% oxygen. For exercise and control lysates, samples were subjected to an increasing oxygen series of 1, 2, 4, 8, 16 and 21% after which the sample was again subjected to 0% oxygen. Gas flow was controlled via gas mass flow controllers and was humidified prior to being exposed to the sample. Gas flows were calibrated daily using a high-resolution gas flow meter (Definer 220, Mesa Labs). Sample drift was corrected using the slope of the linear decline from the initial to final high (21%) and low (0%) measurements. Absorbance at each O_2_ level was recorded as Hb-O_2_ saturation using the wavelength of 436 nm against an isosbestic point of 390 nm (Negrete Jr *et al.*, 2022). Saturation values for each lysate and oxygen level were used to create Hill plots for the determination of P_50_ and Hill coefficients (n_50_; an indicator of Hb cooperativity). The magnitude of the Root effect was determined using the difference in Hb-O_2_ saturation at 100% O_2_ for control/exercise hemolysates versus the pH 8 lysate. OECs for each sample were then redrawn using the sample-specific Root effect value at 100% O_2_ and the equation Hb-O_2_ = pO_2_^n50^/(P_50_^n50^ + pO_2_^n50^) (Barlow *et al.* 2017).

### Analytical methods

Haematocrit (Hct) was determined using heparinized capillary tubes filled with 5 μl of whole blood and centrifuged for 1 min (StatSpin MP). Remaining blood was centrifuged for 2 min at 10 000 g to separate RBCs from plasma. Plasma pH and osmolality were tested (see below) after which the plasma was flash frozen and stored at −80°C. RBCs were washed three times with cold isotonic saline and assessed for intracellular pH (pH_i_) and Hb concentration. Plasma pH_e_ and RBC pH_i_ were measured in a thermostated bath (26°C) using a micro-pH electrode (Accumet, Fisher Scientific). RBC were subjected to two freeze thaw cycles to lyse cells prior to measurement. Haemoglobin concentration was determined using the Drabkin’s colorimetric assay (Drabkin and Austin, 1935) as performed using a plate spectrophotometer, and mean corpuscal haemoglobin concentration (MCHC) was subsequently calculated using Hb concentration and Hct. Plasma osmolality was determined using a vapour pressure osmometer (Wescor; VAPRO 5520). Plasma and tissue lactate, tissue phosphocreatine (PCr), and tissue adenosine triphosphate (ATP) concentrations were measured following the enzymatic methods of Lowry and Passonneau (1972). The muscle was processed according to the procedure described by Booth *et al.* (1995). Anaerobic energy expenditure for each recovery time point using the following formula: (1.5*Δlactate) + ΔATP + ΔPCr, where Δ is the difference between non-exercised and exercised fish (Kieffer, 2000).

### Statistics

For all data, a Shapiro–Wilk test was used to verify normality and a Brown-Forsythe test was used to assess equal variance. Behaviour analysis of air breathing under varying levels of oxygen saturation was completed using a repeated measure one-way analysis of variance (ANOVA) following square root transformation to address normality. Behavioural analysis of air breathing under hypoxia and/or following exercise was completed using a repeated measures two-way ANOVA. Physiological and biochemical analyses investigating recovery period on tarpon were analysed using a one-way ANOVA, or if assumptions of normality and equal variance were not met (muscle PCr) a Kruskal–Wallis ANOVA on Ranks. Analyses of hemolysate P_50_, Root effect and Hill coefficient were performed using a *t*-test, while analysis of the OEC was performed using a repeated measures two-way ANOVA with PO_2_ and pH as factors. All statistical tests were performed using Sigma Plot version 13, and the fiducial level of significance is *P* ≤ 0.05.

## Results

### Air-breathing behaviour in response to hypoxia and exercise

**Figure 1:**
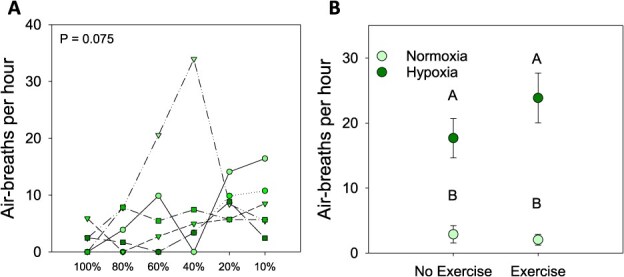
The effects of hypoxia and exhaustive exercise on air-breathing behaviour in Atlantic tarpon (*M. atlanticus*). Panel **A** shows the effects of progressive 25-min bouts of hypoxia (*n* = 6) with symbols showing the responses of individual fish. Panel **B** shows the effects of 60-min bouts of hypoxia following exhaustive exercise or rest (mean ± S.E.M.; *n* = 6). Letters in panel B denote significant differences as determined by repeated measures two-way ANOVA

Series 1 examined air breathing using a stepwise (24.2 ± 0.8 min per step) hypoxia protocol, which revealed no significant difference in breathing rate, despite the fact that a trend toward increased breathing below 40% O_2_ saturation was observed (Fig. 1a). A second trial (Series 2; Fig. 1b) exposed fish normoxia or 20% O_2_ saturation for 1 h, after which fish were exercised to exhaustion and observed for a second 1 h period. This experimental design revealed that Atlantic tarpon significantly increased air breathing in hypoxia as compared to normoxia (RM two-way ANOVA; *F*_1,5_ = 390.7, *P* < 0.001); however, there was no significant increase in air-breathing post-exercise in either normoxia or hypoxia (RM two-way ANOVA; *F*_1,5_ = 1.12, *P* = 0.324) and there was no significant interaction between oxygen level and exercise status (*P* = 0.47).

### Biochemical recovery from exhaustive exercise

**Figure 2:**
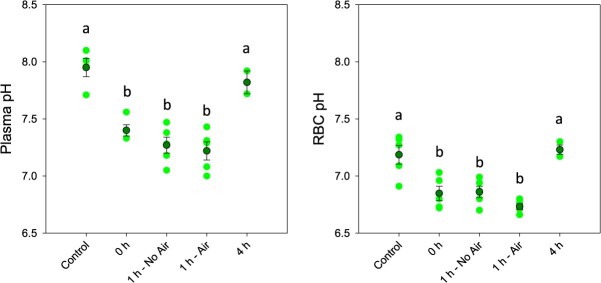
Plasma and red blood cell intracellular pH following exhaustive exercise and recovery with or without access to air. Dark green symbols represent the mean ± S.E.M., while light green symbols show the individual data points. Letters denote significant differences as determined by one-way ANOVA (*P* ≤ 0.05; *n*: control = 5, 0 h = 5, 1 h no air = 5, 1 h air = 5, 4 h = 2)

**Figure 3:**
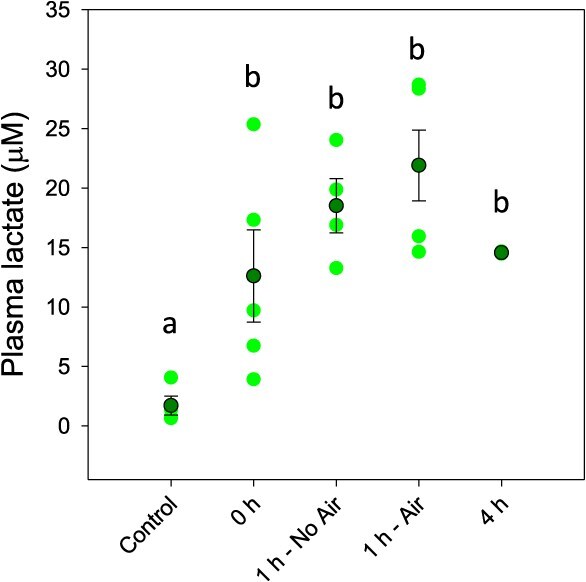
Plasma lactate following exhaustive exercise and recovery with or without access to air. Dark green symbols represent the mean ± S.E.M., while light green symbols show the individual data points. Letters denote significant differences as determined by one-way ANOVA (*P* ≤ 0.05; *n*: control = 4, 0 h = 5, 1 h no air = 4, 1 h air = 5, 4 h = 2)

The effects of exhaustive exercise and the time course of recovery in Atlantic tarpon was assessed using a suite of plasma and muscle metabolites. In the plasma, evidence of exhaustive exercise stress was observed immediately post-exercise in the form of significantly reduced plasma pH (*F*_4,20_ = 28.465; *P* < 0.001) and red blood cell pH_i_ (*F*_4,20_ = 17.119; *P* < 0.001) (Fig. 2), as well as significantly elevated plasma lactate (*F*_4,18_ = 12.769; *P* < 0.001) (Fig. 3). There was no evidence of recovery of any of the three measures following 1 h, but both plasma pH and red blood cell pH_i_ fully recovered by 4 h post-exercise. Plasma lactate remained significantly elevated following 4 h of recovery; however, the levels had declined by approximately 33% relative to the peak response. There was no significant effect of removing access to air on recovery of any blood measure at the 1 h time point. There was no significant effect of exercise on Hct, MCHC and plasma osmolality at any time point relative to the no-exercise controls. Total blood Hb concentration exhibited a post-exercise decline, but this was only significant relative to the 4 h recover time point (Table 1).

**Table 1:** The effects of exhaustive exercise and air access on recovery of haematological characteristics of Atlantic tarpon (*M. atlanticus*)

	**Control**	**0-h**	**1-h air**	**1-h no air**	**4-h**
Osmolality (mOsm)	292 ± 9	307 ± 15	314 ± 1	301 ± 14	301 ± 1
Hct (%)	40.7 ± 5.4	30.8 ± 3.1	33.4 ± 2.1	37.9 ± 1.5	35 ± 3
MCHC (mM)	5.0 ± 0.5	6.3 ± 0.5	4.6 ± 0.5	4.8 ± 0.3	12.2 ± 2.8
Hb_4_ (mM)	2.9 ± 0.6 ^ab^	1.6 ± 0.3 ^a^	1.5 ± 0.1 ^a^	1.8 ± 0.1 ^a^	4.3 ± 0.2 ^b^

**Figure 4:**
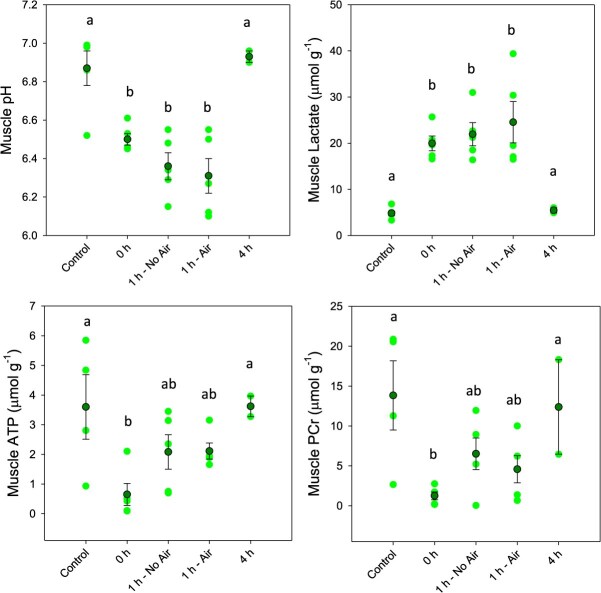
Muscle pH, lactate, ATP and PCr following exhaustive exercise and recovery with or without access to air. Dark green symbols represent the mean ± S.E.M., while light green symbols show the individual data points. Letters denote significant differences as determined by one-way ANOVA (*P* ≤ 0.05; *n*: control = 4–5, 0 h = 5, 1 h no air = 4–5, 1 h air = 5, 4 h = 2)

**Figure 5:**
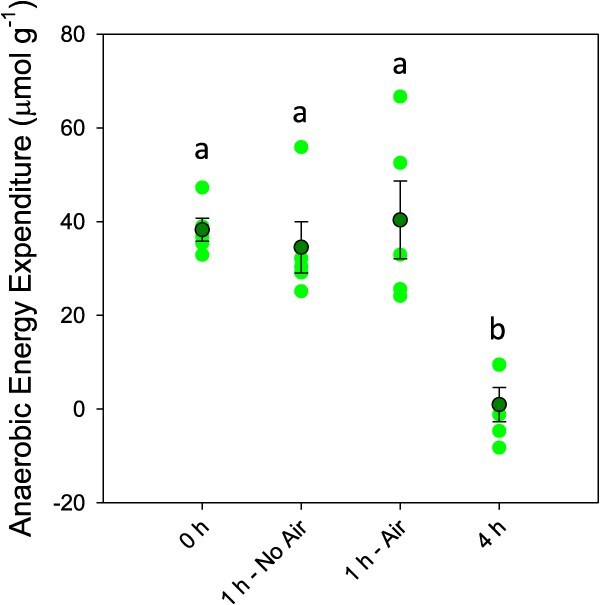
Anaerobic energy expenditure following exhaustive exercise and recovery with or without access to air. Dark green symbols represent the mean ± S.E.M., while light green symbols show the individual data points. Letters denote significant differences as determined by one-way ANOVA (*P* ≤ 0.05; n: control = 4–5, 0 h = 5, 1 h no air = 4–5, 1 h air = 5, 4 h = 2)

In the white muscle tissue, exhaustive exercise caused immediate responses in pH_i_ as well as lactate, ATP and PCr concentrations (Fig. 4). Muscle pH showed an immediate ΔpH of 0.36 units (*F*_4,20_ = 15.823; *P* < 0.001), which decreased further to ΔpH of 0.53 after 1 h of recovery. Muscle lactate (*F*_4,20_ = 14.641; *P* < 0.001), PCr (*F*_4,19_ = 6.004; *P* = 0.003) and ATP (*F*_4,19_ = 5.967; *P* = 0.003) also showed peak responses immediately post-exercise, with ATP and PCr showing statistical evidence of recovery at the 1 h time point. Muscle lactate was unchanged from immediate post-exercise values following 1 h of recovery. The combined anaerobic exercise expenditure following exercise was calculated (Fig. 5), which showed that peak expenditure occurred immediately following exercise, but showed little recovery following 1 h of rest. All four measures of exercise stress in white muscle returned to control levels by 4 h post-exercise, which corresponded with a return of anaerobic energy expenditure to zero.

### Effects of exercise on haemoglobin function

**Figure 6:**
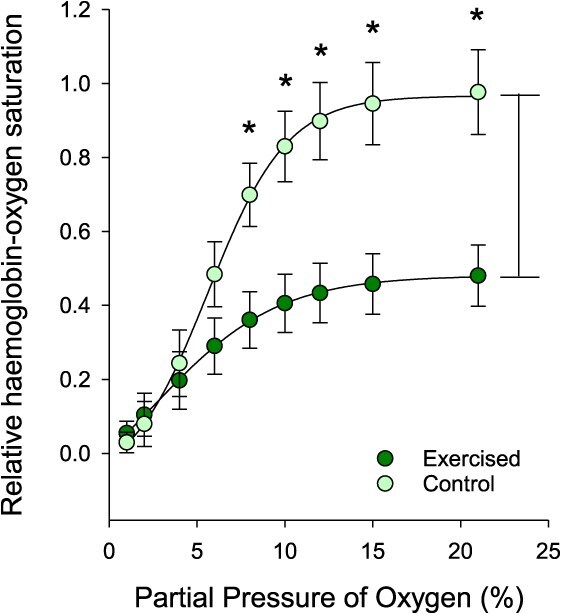
Mean oxygen equilibrium curve of Atlantic tarpon hemolysates from control or exercised (0-h) individuals. Data (mean ± S.E.M.; *n* = 4) are expressed relative to the control at 21% oxygen. Asterisks denote a significant difference in relative Hb-O_2_ saturation between control and exercised as determined by two-way ANOVA

The observation that Atlantic tarpon exhibited a prolonged depression in red blood cell pH_i_ raised the potential that the oxygen supply cascade of recovering animals could be compromised. To explore this possibility, we generated OECs (Fig. 6) from red cell homogenate preparations from control fish and immediately post-exercise fish that were representative of endogenous conditions (i.e. pH and NTP:Hb ratios). There was no difference between control fish (hemolysate pH = 7.2) and exercised fish (hemolysate pH = 6.8) P_50_, and there was no difference in the calculated Hill coefficient (Table 2); however, there was a significant difference in the magnitude of the Root effect in the respective samples—as defined by the change in Hb-O_2_ saturation at 21% PO_2_ relative to pH 8.2. When the OECs were redrawn for each sample to incorporate the Root effect and normalized to the average control Hb-O_2_ saturation, a clear difference between control and exercised hemolysates was observed (Fig. 6). Specifically, there was a significant effect of treatment (*F*_1,48_ = 8.944; *P* = 0.024) and PO_2_ (*F*_8,48_ = 46.26; *P* < 0.001) on Hb-O_2_ saturation, as well as an interaction between the two factors (*F*_8,48_ = 7.965; *P* < 0.001), which showed that exercised fish exhibited a significant reduction in relative Hb-O_2_ saturation at PO_2_ values above 6% PO_2_.

**Table 2:** Haemoglobin oxygen affinity characteristics for Atlantic tarpon (*M. atlanticus*)

	**Control**	**Exercised**
P_50_ (mmHg) ^a^	43.6 ± 6.5	37.9 ± 7.8
n_50_	3.23 ± 0.64	2.08 ± 0.57
Root Effect (% sat.) ^b^	34 ± 6	70 ± 8
Hemolysate pH	7.27 ± 0.01	6.93 ± 0.02

a Not corrected for Root effect.

b Relative to pH 8.

## Discussion

The first goal of this study was to assess whether the air-breathing behaviour of Atlantic tarpon is obligate or facultative. The best available evidence to date suggests that Atlantic tarpon are facultative air breathers, which was based on the observation that tarpon were able to survive in lab holding conditions without access to air for 1 to 2 weeks (Geiger *et al.*, 2000). While this is convincing, there remains some uncertainty owing to fact that animal metabolism and condition were not simultaneously assessed. As such, we performed a series of hypoxia exposure trials to validate that Atlantic tarpon are facultative air breathers. By definition, facultative air-breathing fish exhibit differences in air-breathing frequency in normoxia and hypoxia; they can physiologically meet oxygen requirements via their gills in normoxia but air-breathe to obtain oxygen under hypoxic conditions (Graham, 1997). Our first experimental series exposed fish to progressive hypoxia in 25-min intervals, and while no significant increases in air-breathing rate occurred throughout the trial, there did appear to be a trend toward increased air breathing as O_2_ saturation decreased. As such, a second series exposed fish to 20% air saturation for 1 h, which demonstrated that Atlantic tarpon significantly increased their air-breathing rate under hypoxia compared to normoxia. These findings agree with the suggestion put forth by Geiger *et al.* (2000), and are also congruent with the known facultative air-breathing behaviour of Pacific tarpon (Seymour *et al.*, 2004).

While Atlantic tarpon are clearly a facultative air breather with respect to hypoxia, and thus are not in danger of suffocating post-exhaustive exercise, they may still benefit from air breathing with respect to metabolic recovery. In fact, this has been shown previously for Indo-Pacific tarpon, at least with respect to plasma lactate (Wells *et al.*, 2003). In general, exhaustive exercise is powered by anaerobic swimming that is associated with a sharp decline in energy stores (e.g. ATP, PCr and glycogen) in the white muscle (Wood, 1991; Kieffer, 2000). Anaerobic swimming also leads to lactate and metabolic proton build-up in the muscle, which “spills over” into the plasma (Wood, 1991; Kieffer, 2000; Negrete Jr *et al.*, 2024). These biochemical impacts of exercise must be corrected for fish to return to normal activity, which is commonly referred to as an oxygen debt. The oxygen debt leads to the commonly observed elevated post-exercise oxygen consumption rates that can range from minutes to hours (Lee *et al.*, 2003; Johansen and Esbaugh, 2017). Presumably the much greater oxygen content in air would facilitate quicker recovery, and thus we hypothesized that post-exercise tarpon would significantly increase air-breathing rate regardless of aquatic oxygen status. In fact, multiple studies have shown exercise can stimulate air breathing during or after exercise (Burleson *et al.*, 1998; Farmer and Jackson, 1998). However, when Atlantic tarpon were chased to exhaustion and allowed to recover for 1 h in either normoxia or hypoxia, only those fish recovering in hypoxia increased their air-breathing rate. There was also no significant difference in air-breathing rate for non-exercised hypoxia exposed and post-exercise hypoxia exposed fish. This suggests that exhaustive exercise does not stimulate air breathing in Atlantic tarpon.

The first two series of experiments demonstrated that Atlantic tarpon are not obligate air-breathers, nor does exercise stimulate air-breathing behaviour. But, this did not show that air breathing is irrelevant for exercise recovery in Atlantic tarpon. Note that exercised fishes maintained a baseline level of air breathing that was equal to control fishes. Importantly, our observational tanks were also relatively shallow as compared to the typical habitat of Atlantic tarpon. As such, we performed a third experiment to track the metabolic recovery of Atlantic tarpon following an exhaustive exercise event, and test the hypothesis that access to air would provide a tangible benefit to Atlantic tarpon during recovery from exhaustive exercise.

The general patterns of exercise metabolite disruption following exhaustive exercise followed the profiles described for other studied teleosts, including rainbow trout (*Oncorhynchus mykiss*)(Milligan and Wood, 1986a, 1986b, 1987; Ferguson *et al.*, 1993; Kieffer *et al.*, 1994), Atlantic salmon (*Salmo salar*) (Tufts *et al.*, 1991; Wakefield *et al.*, 2004), large-mouth bass (*Micropterus salmoides*) (Suski *et al.*, 2006; Gingerich and Suski, 2012) and red drum (*Sciaenops ocellatus*)(Martin *et al.*, 2023) amongst others (Suski *et al.*, 2007; Kieffer, 2010). Briefly, the white muscle tissue showed a significant increase in anaerobic energy expenditure immediately following exercise that was the product of significant reductions in ATP and PCr concentrations, as well as a significant elevation in lactate concentration that was presumably paralleled by a reduction of muscle glycogen. The magnitude of the responses immediately post-exercise generally aligned with those of other species, being slightly lower than salmonids (Kieffer, 2000), red drum (Martin *et al.*, 2023) and Indo-Pacific tarpon (Wells *et al.*, 2003) but higher than largemouth bass (Suski *et al.*, 2006), with the obvious caveat that body size is known to impact the magnitude of such responses (Ferguson *et al.*, 1993; Kieffer *et al.*, 1996; Gingerich and Suski, 2012; Martin *et al.*, 2023). Similarly, the blood showed a significant elevation of plasma lactate concentration as well as a significant decline in plasma and red blood cell pH. Interestingly, the change in plasma lactate immediately post-exercise was 12.6 ± 3.9 mM, which is approximately double what is typically reported as a consequence of exhaustive exercise (Milligan and Girard, 1993; Kieffer *et al.*, 1994; Suski *et al.*, 2006; Suski *et al.*, 2007; Currey *et al.*, 2013; Martin *et al.*, 2023), including values following a 15-min angling event in Indo-Pacific tarpon (Wells *et al.*, 2003). Tarpon plasma also exhibited a ΔpH in the plasma that was on the higher end of what it is typically observed, which has generally ranged from −0.35 to −0.5 pH units (Milligan and Wood, 1986a; Currie and Tufts, 1993; Kieffer *et al.*, 1994; Li *et al.*, 2010), although red drum showed a smaller effect of only −0.1 units (Martin *et al.*, 2023). More notable was the change of intracellular red blood cell pH, which showed a ΔpH of −0.35 units, which is nearly double what has been previously reported for rainbow trout (Currie and Tufts, 1993).

The overall pattern of recovery following exhaustive exercise showed white muscle ATP and PCr exhibiting the earliest signs of recovery after only 1 h, while muscle lactate and pH_i_ as well as plasma pH and red blood cell pH_i_ all recovered to control values by 4 h post-exercise. Plasma lactate was the slowest parameter to recover, as it had not yet returned to control levels by 4 h post-exercise; however, this pattern is relatively common in fishes (Omlin and Weber, 2013). Literature patterns for recovery are quite variable and are known to be impacted by temperature (Kieffer, 2000). For example, cold water (15°C) rainbow trout exhaustively exercised did not show full recovery of muscle and plasma lactate until 12 h post-exercise. Conversely, warm water species similar to Atlantic tarpon, such as bonefish and red drum, generally exhibited full recovery within 4 h (Suski *et al.*, 2007; Martin *et al.*, 2023). An important caveat to the current study is that the 4 h post-exercise time point has a low sample size, and thus trends at this timepoint should be viewed with caution. Most important to the current study, no recovery parameter was influenced by restricted access to air during the recovery period. This provides further evidence that air breathing provides no tangible benefit to Atlantic tarpon following the exhaustive exercise events that are incurred during recreational angling. Interestingly, this work stands in contrast to previous findings from the Indo-Pacific tarpon that showed that access to air significantly reduced the plasma lactate load at 1 h post-exercise (Wells *et al.*, 2003).

The sustained reduction in red blood cell pH_i_ raised the possibility that oxygen transport capacity in this species may be compromised post-exercise. This observation is relatively well documented in teleosts whereby exercise induced acid–base disturbances reduce red blood cell pH, and thereby reduce the Hb-O_2_ binding capacity in accordance with the Root effect (Milligan and Wood, 1987; Tufts, 1991, Tufts *et al.*, 1991). The presence of a strong Root effect means that even high oxygen partial pressures will not result in the ability of Hb to bind additional oxygen at low pH (Root, 1931). While we did not directly measure oxygen content of the blood post-exercise, our data nonetheless suggest that Atlantic tarpon will experience a significant reduction in blood oxygen carrying capacity owing to the particular characteristics of the Root effect. For Atlantic tarpon, control red blood cell pH_i_ resulted in 70 ± 8% saturation relative to a theoretical maximum determined at an alkaline pH 8. More importantly, the exercised samples with significantly reduced pH exhibited a mean 34 ± 6% saturation, or half that of the controls. The magnitude of the Root effect described is similar to that shown for rainbow trout (Rummer *et al.*, 2010). Such an effect would be beneficial for the Root-effect-mediated oxygen extraction at the tissues that is thought to benefit exercise performance (Rummer *et al.*, 2013; Randall *et al.*, 2014; Rummer and Brauner, 2015; Dichiera and Esbaugh, 2020; Harter *et al.*, 2024). However, it appears that Atlantic tarpon do not initiate a strong red blood cell pH_i_ protection response, which would lead to compromised oxygen uptake at the respiratory epithelium and likely result in slower recovery from exercise. In fact, this may partially explain why air breathing is not utilized following exercise recovery. In essence, the lack of red blood cell pH_i_ recovery combined with the characteristics of the Root effect mean that the extra oxygen that could be obtained through air breathing is not bioavailable. It is also interesting to note that Atlantic tarpon do not significantly elevate haematocrit or total Hb following exercise, which is a common approach to improve oxygen carrying capacity of the blood (Gallaugher and Farrell, 1998). This again stands in contrast to the Indo-pacific tarpon (Wells *et al.*, 2003) and demonstrates how even closely related species can diverge in their approach to exercise recovery.

In conclusion, this study has demonstrated that Atlantic tarpon are facultative air-breathers that do not use air breathing to benefit exercise recovery, setting them apart from the more well studied Indo-Pacific tarpon. In fact, Atlantic tarpon recovery patterns mirror those of other well-studied species, particularly those studied at a similar temperature profile. An important caveat of this work is that the animal sizes are far below the prized catches of elite anglers, and thus some caution is needed owing to possible allometric relationships. For example, many sportfish have been shown to exhibit greater anaerobic energy expenditures in larger sized individuals as compared to small individuals (Ferguson *et al.*, 1993; Gingerich and Suski, 2012; Martin *et al.*, 2023). As such, it cannot be ruled out that air breathing may become more important for exhaustive exercise recovery in larger sized animals. Nonetheless, this work provides important validation that specialized CAR protocols are likely not needed to address the unique air-breathing physiology of Atlantic tarpon, and thus managers can focus on the most effective ways to limiting stress during CAR event: minimized fight time, handling and air exposure while quickly removing hooks (Cooke *et al.*, 2013).

## Data Availability

All data will be made available upon request to the corresponding author.
